# Revisiting Risk Governance of GM Plants: The Need to Consider New and Emerging Gene-Editing Techniques

**DOI:** 10.3389/fpls.2018.01874

**Published:** 2018-12-21

**Authors:** Sarah Z. Agapito-Tenfen, Arinze S. Okoli, Michael J. Bernstein, Odd-Gunnar Wikmark, Anne I. Myhr

**Affiliations:** ^1^GenØk - Centre for Biosafety, SIVA Innovation Centre, Tromsø, Norway; ^2^Unit for Environmental Science and Management, North West University, Potchefstroom, South Africa

**Keywords:** genetically modified plants, crop breeding, risk assessment, CRISPR (clustered regularly interspaced short palindromic repeats)/Cas9, transgenic plants

## Abstract

New and emerging gene-editing techniques make it possible to target specific genes in species with greater speed and specificity than previously possible. Of major relevance for plant breeding, regulators and scientists are discussing how to regulate products developed using these gene-editing techniques. Such discussions include whether to adopt or adapt the current framework for GMO risk governance in evaluating the impacts of gene-edited plants, and derived products, on the environment, human and animal health and society. Product classification or definition is one of several aspects of the current framework being criticized. Further, knowledge gaps related to risk assessments of gene-edited organisms—for example of target and off-target effects of intervention in plant genomes—are also of concern. Resolving these and related aspects of the current framework will involve addressing many subjective, value-laden positions, for example how to specify protection goals through ecosystem service approaches. A process informed by responsible research and innovation practices, involving a broader community of people, organizations, experts, and interest groups, could help scientists, regulators, and other stakeholders address these complex, value-laden concerns related to gene-editing of plants with and for society.

## Introduction

New and emerging gene-editing techniques being developed include clustered regularly interspaced short palindromic repeats (CRISPR), oligonucleotide directed mutagenesis (ODMs), meganucleases (EMNs), zinc finger nucleases (ZFNs), and transcription activator-like effector nucleases (TALENs). These new techniques open the possibility for editing genetic information and modulating gene expression in organisms in faster and more targeted ways. Gene-editing techniques raise the possibility of targeting, *in vivo*, a specific gene or sequence in the genome of virtually any species. Targeted gene modification can be the deletion, insertion or alteration of nucleotides in an existing molecule of DNA or RNA, as well as insertions or deletions of large sequences in specific target locations.

Regulators and scientists discuss whether gene-edited organisms should be subjected to the same risk assessment and management requirements as genetically modified organisms (GMOs). In general terms, GMOs require regulatory approval before environmental release and use in food and feed. Regulatory approval is informed by an assessment of risks to human health and the environment. An open question is thus whether and how the EU current framework applying to GMOs needs to be applied, adapted, and updated for new and emerging gene-editing techniques.

In this paper, we discuss the potential challenges new and emerging gene-editing techniques pose to established risk governance strategies. We focus on regulatory requirements for assessing health and environmental risks as established under EU Directives, and elaborate how biosafety research can strengthen risk assessment (RA) and management. At present, national frameworks in the EU Member States are transposing the EU-level framework laid out by the respective EU Directives and thus are harmonized with the general community framework. There are challenges with traceability and monitoring of products developed using new and emerging gene-editing techniques. In addition, risk assessment and management of genetically modified (GM) plants is constrained by limitations in transparency regarding public disclosure related to product development. We propose that the framework of responsible research and innovation (RRI) offers a useful way to improve GM risk governance research and practice for biosafety of crop development with new and emerging gene-editing techniques.

## Overview of the Regulatory Landscapes for GMOs

### The Scope of Current GMO Regulation

**FIGURE 1:**
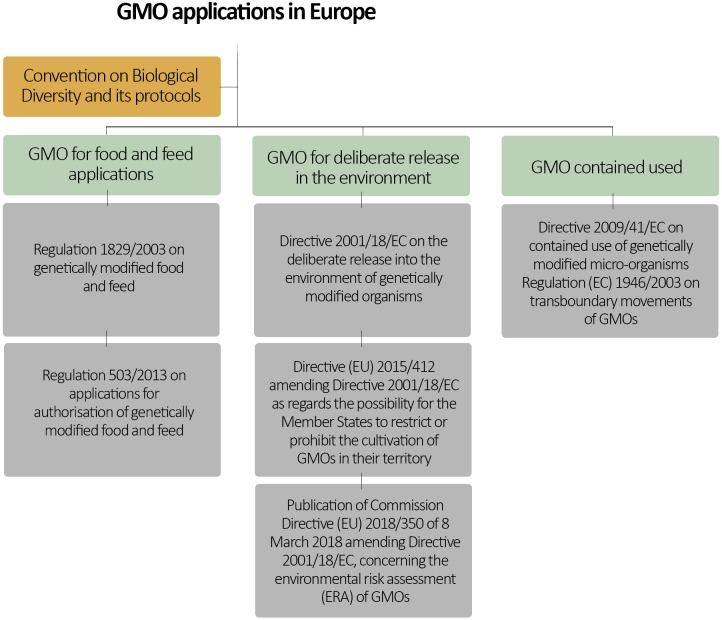
European regulatory framework for GMOs. Note that European Union is also a signatory to the United Nations Convention on Biological Diversity and its related protocols. Therefore, it can be also considered as part of the European GMO regulatory framework.

In considering challenges with risk governance of new and emerging gene-editing techniques, it is instructive to start with current regulations related to GMOs. European regulatory requirements that address environmental release of GMOs and of GM foods and feeds are established in EU Directive 2001/18/EC (originally 90/220/EC), in regulation (EC) No. 1829/2003 and its sister regulations (Figure 1), as well as in various national frameworks. Central to any regulatory requirement is an element of assessing risks to human, animal, and environmental health. At the pan-European-level, such risk assessments are based on a case-by-case approach and a stepwise procedure. The European Food Safety Authority (EFSA) provides scientific review and assessment of safety and environmental impact of GMOs, while the European Commission is responsible for risk management decisions.

Other countries, for example the United States, have not developed a new regulatory process for GMOs or gene-edited organisms. In the United States, depending on the genetic modification and the host organism, one or several United States federal agencies would be involved in GMO regulation, for example the Food and Drug Administration (FDA), the Environmental Protection Agency (EPA) and the Animal and Plant Health Inspection Service (APHIS) (Schuttelaar and Partners, 2015; Ishii and Araki, 2017). In the United States, gene-edited plants are not subject to specific regulatory requirements unless they have novel traits expressing, for example, herbicide tolerance or antibiotic resistance. Thus, for the United States framework not all novel traits trigger regulatory oversight, but only a defined range of traits of specific concern, e.g., compositional differences that are not GRAS, pesticidal properties or traits and genetic elements derived from organisms which are plant pathogens or that may induce plant pathogenicity.

Similarly, Canada has proceeded without developing new, GMO-specific regulatory requirements and uses already adopted regulatory frameworks. In Canada, GMOs fall under consideration of “plant with novel trait” a category which includes not only GMOs, but also plants with induced mutations, natural mutations, and exotic germplasm not previously grown in Canada (Smyth, 2017). The United States and Canadian regulatory frameworks focus primarily on human safety and environmental risk, the efficacy of the novel trait, and the intended use of the product. By contrast, other countries, for example, Norway, consider non-safety-related aspects of GMOs such as socio-economic considerations, ethical issues, and potential contribution to sustainable development (see the Norwegian Gene Technology ACT, 1993)^1^.

Where Canada has adopted a product-based regulatory system and the United States has a hybrid system, Argentina and Europe have a process-based system. By adopting a process-based system, GMOs are regulated differently than other products (e.g., organisms and plants developed by other methods than GM technology) and according to a specific regulatory framework: this is the case in Europe. Those who argue against novel regulation to new and emerging gene-editing techniques object on the grounds of a product-based system of regulation. A main argument of this group is the final product—gene-edited organisms— contain comparable types of genetic changes (or mutations) to organisms originating from established methods of genetic modification, such as random mutagenesis techniques (e.g., irradiation).

Reviewing the regulatory landscape for GMOs reveals how fundamentally different approaches to regulation may continue, independent of the regulatory system the country has adopted, to divide national responses to risk governance of new and emerging gene-editing techniques. Based on this observation, Ishii and Araki (2017) argue for international efforts of regulatory harmonization, for example by the Cartagena Protocol on Biosafety (CP). At the international level, the Convention on Biological Diversity (CBD) has served as the umbrella treaty for the CP since 2000 (entry into force in 2003). The CP agreement aims to ensure the safe handling, transport, and use of living modified organisms (LMOs) resulting from modern biotechnology, taking into account possible adverse effects on biological diversity as well as risks to human health. The CP treaty offers a benchmark and guide for many developing countries exploring adoption of GMO regulatory frameworks. Further, the CBD and CP have established interactive platforms for sharing information and knowledge about international biosafety issues, including unintentional transboundary movement of LMOs and emergency measures for unauthorized GMO escape.^2^ Under the CP treaty, organisms altered with new and emerging gene-editing techniques would seem to fall under the agreement for safe handling, transport and use of LMOs—indeed, the LMO definition was left intentionally open to remain relevant for precisely such future developments (Mackenzie et al., 2003). The treaty has been signed and ratified by some 170 countries, including the EU and Norway. Several countries, however, including Russia, United States, Canada, and Argentina are not parties to the CP, which may hamper any international effort of regulatory harmonization.

The first country to adopt regulation specifically for new and emerging gene-editing techniques was Argentina (Resolution No.173/2015). Argentina has issued a Resolution which specifies criteria to assess whether certain products are covered by the definitions included in their biosafety law. An important criterion is whether a product contains a “novel combination of genetic material” (Whelan and Lema, 2015). Brazil issued a similar resolution earlier this year (Resolution No. 16/2018), which includes a criterion to determine the regulatory status of new and emerging gene-editing techniques, for example if products using these techniques will be considered a GMO as per Brazilian Biosafety Law. Despite these early actions, most countries in Europe and elsewhere, and at international levels (e.g., European Union, OECD, CBD, the CP) are still discussing whether and how to adapt GMO risk governance frameworks to account for new and emerging gene-editing techniques.

### Regulatory Challenges for New and Emerging Gene-Editing Techniques

National responses to the growing use of new and emerging gene-editing techniques in plants raise questions of whether such developments (a) might be exempt from current GMO regulations, and/or (b) if existing regulations require revision and adaptation to appropriately manage new techniques and resulting products (Wolt et al., 2016). As noted above, the main argument for exemption from current GMO regulation is the similarity of organisms altered with new and emerging gene-editing techniques to organisms originating from random mutagenesis (e.g., irradiation). The argument of exemption based on similarity posits that gene-edited organisms are indistinguishable from products created by already exempted processes (Jones, 2015b; Davison and Ammann, 2017). A central assumption of this argument is that any risks associated with new and emerging techniques for gene-editing will also be similar and equal to, or less significant than risks associated with exempted techniques or products (Hartung and Schiemann, 2014; Sprink et al., 2016; Globus and Qimron, 2018).

The EFSA GMO Panel opinion addressing the safety assessment of plants developed using Zinc Finger Nuclease and other Site-Directed Nucleases with similar function (EFSA Panel on Genetically modified organisms (GMO), 2012) and the Institute for Prospective Technological Studies and Institute for Health and Consumer Protection (both from the Joint Research Centre at the European Commission) (Lusser et al., 2011) have set forth three major categories of new and emerging gene-editing techniques (Box 1).

**BOX 1. European authorities’ definition and categorization of gene-editing techniques.**

Site-Directed Nucleases-1 (SDN-1) generates site-specific random mutations (changes of single base pairs, short deletions and insertions) by non-homologous end-joining. During SDN-1, no repair template is provided to the cells together with the SDN. Therefore, in the case of insertions, the inserted material is derived from the organism’s own genome, i.e., it is not exogenous.

Site-Directed Nuclease-2 (SDN-2) generates site-specific desired point mutation by DNA repair processes through homologous recombination (specific nucleotide substitutions of a single or a few nucleotides or small insertions or deletions). During SDN-2, an exogenous DNA template is delivered to the cells simultaneously with the SDN for achieving desired nucleotide change via homology dependent repair.

Site-Directed Nuclease-3 (SDN-3) targets delivery of transgenes (insertions) by homologous recombination. Exogenous DNA fragments or gene cassettes up to several kilo base pairs (kbp) in length can be inserted to a desired site in the genome or a gene.

European Food Safety Authority holds that products developed using SDN-3 techniques would be categorized as GMOs and regulated under EU Directive. There has been a disagreement as to whether products arising from use of SDN-1 or SDN-2 might be exempt (Sprink et al., 2016). For example, while waiting for a decision from the European Court of Justice (EJC), Sweden decided that gene-editing products with no recombinant DNA insertions may (e.g., SDN-1), on a case-by-case basis, be exempted from GMO regulation (Nature Editorial, 2017). The recent EJC ruling,^3^ however, now clarifies that all SDN techniques fall under the EU Directive.

The scope of the EU legislation and Article 2(2) of the Release Directive (Directive 2015/412 amending Directive 2001/18/EC) provide the definition of a GMO. These laws define a GMO as, “An organism, with the exception of human beings, in which the genetic material has been altered in a way that does not occur naturally by mating and/or natural recombination.” In Annex IA, part 1 scopes techniques of genetic modification, stating:

“Techniques of genetic modification referred to in Article 2(2)(a) are inter alia [not an exhaustive list]: recombinant nuclei acid techniques involving the formation of new combinations of genetic material by the insertion of nucleic acid molecules produced by whatever means outside an organism, into any viruses, bacterial plasmid or other vector system and their incorporation into a host organism in which they do not naturally occur but in which they are capable of continued propagation; techniques involving the direct introduction into an organism of heritable material prepared outside the organism including micro-injection, macro-injection and micro-encapsulation; cell fusion (including protoplast fusion or hybridization techniques where live cells with new combinations of heritable genetic material are formed through the fusion of two or more cells by means of methods that did not occur naturally.”

Whereas Article 3 and Annex IB specifies exemptions to the Directive (Zetterberg and Björnberg, 2017). Excluded techniques include mutagenesis and cell fusion, including protoplast fusion, of plant cells of organisms which can exchange genetic material through traditional breeding. Annex IB lists techniques that do produce a GMO under the Directive but are exempt on the condition that they do not involve the use of recombinant nucleic acid molecules or GM organisms other than those produced by one or more of the techniques/methods listed in Annex IB.

In summary, the EU Directive provides grounds for the argument for exemption of gene-edited plants due to the potential similarity between gene-edited plants and those originating from mutagenesis techniques. However, argument for exemption may be limited because (a) the Directive does not define mutagenesis (Eriksson et al., 2018), and (b) the argument solely lies on the technique used (i.e., mutagenesis). Ambiguity arises because there are a variety of mutagenesis techniques that can be applied (e.g., irradiation, CRISPR, ODM, etc.) and thus, the Directive does not acknowledge whether the process of gene-editing by each of these techniques leads to the formation of an organisms covered by the GMO definition. The use of the term ‘mutagenesis’ may therefore lead to the false impression that there is only one mutagenic technique in place.

Heinemann (2015) argues that the reasoning based upon distinguishability of products and not genetic engineering techniques is not relevant to the Cartagena Protocol or the EU Directive because technique is neither relevant to the definition of a GMO nor to the description of a process by which a GMO is made. Moreover, distinguishability is a function of existing technology. As technologies change, so might the ability to distinguish products from each other. We acknowledge that not all products of new and emerging gene-editing techniques are indistinguishable. For example, in certain cases of multiplexed editing, where edited genes are located in multiple chromosomal sites, or other products where characterization and traceability is possible (e.g., large deletions with SDN-1 techniques) (c.f., Duensing et al., 2018). In the context of debates about regulation of new and emerging gene-editing techniques, however, it seems problematic to be at once a new technique and a technique associated with a long history of safe use. This issue is a core focus of the recent ECJ ruling on the interpretation and validity of Articles 2 and 3 of, and Annexes IA and IB to, Directive 2001/18/EC on the deliberate release into the environment of GMOs.

According to the provisional text of the ECJ ruling, organisms and products of new and emerging gene-editing techniques will fall under GMO Directive. The court is clear that these new techniques, “Alter genetic material of an organism in a way that does not occur naturally” (paragraph 28, page 8 of the ECJ provisional text in English). Moreover, the ECJ opinion draws on the fact that these new techniques are not like “those which have conventionally been used in a number of applications and have a long safety record” (paragraph 26, page 8 of the ECJ provisional text in English). Instead of focusing on how mutagenesis techniques might produce “undistinguishable” products, the ECJ viewpoint is that it is impossible to determine with certainty the existence and extent of risks presented by new directed mutagenesis techniques without a premarket RA. The ruling further states, “For the purpose of interpreting a provision of EU law, it is necessary to consider not only its wording but also the context in which it occurs and the objectives pursued by the rules of which it is part” (paragraph 42, page 9 of the ECJ provisional text in English). The ECJ further reiterated the precautionary principle which was taken into account in the drafting of the directive and so also must be taken into account in implementation.

## Suitability of Current Risk Governance of GMO Plants

### Current Guidance on Risk Assessments of GMOs Under European Regulation

**FIGURE 2:**
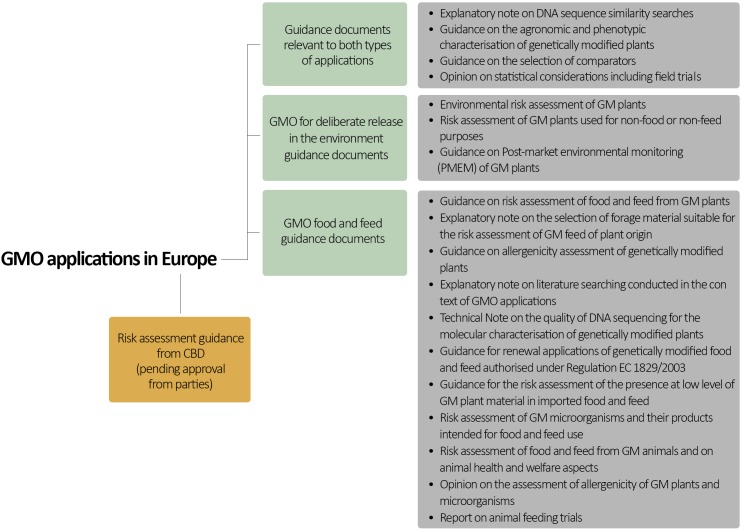
European scientific guidelines for GMO risk assessment. Note that this is not an exhaustive list of all relevant guidance documents.

Guidance for evaluating the impact of genetically modified (GM) plants and plant-derived products in the EU is provided by two documents based on Directive 2001/18/EC and Regulation (EC) No. 1829/2003: guidance on Environmental Risk Assessment (ERA) of GM plants (EFSA Panel on Genetically modified organisms (GMO), 2010) and guidance for risk assessment of food and feed (RAFF) derived from GM plants (EFSA Panel on Genetically modified organisms (GMO), 2011; Figure 2) (Box 2). The ERA focuses mainly on the impact of GMOs on the environment, including humans and animals as components of the environment. By contrast, RAFF focuses only on the health of humans and animals upon consumption of GM foods and feeds.

**BOX 2. Steps in ERA of GM plants.**

Problem formulation and hazard identification

In this first step, the assumptions underlying the ERA are explicitly formulated in the form of a problem statement, involving identification of the potentially hazardous characteristics of the GM plant, the nature of the hazards and exposure paths of the environment to harm associated with the hazards. By comparing the GM plant to its non-modified parent (or other appropriate comparators), differences in the GM plants that may constitute harm and their potential environmental consequences can be identified. Quantifiable assessment endpoints and testable hypotheses that will guide data generation and assessment are also defined.

Hazard characterization

During hazard characterization, the environmental harm potentially associated with each identified hazard is evaluated according to the set out hypotheses, and expressed quantitatively and/or qualitatively. In qualitative expression, the categorical terms “high,” “moderate,” “low,” or “negligible” are employed to express the scale of severity of identified hazards.

Exposure characterization

In this step, the likelihood of the adverse effect occurring is estimated. Similar to hazard characterization, “likelihood” is denoted using ordered categorical descriptions of “high,” “moderate,” “low” or “negligible.” Quantitative expression of 0 to 1 can also be used to express likelihood where 0 represents impossibility and 1 represents certainty.

Risk characterization

An estimate of the risk of adverse effect is made for each identified hazard at this stage. This is achieved by combining the magnitude of the consequences of the hazard and the likelihood that the consequences related to the hazard will occur, and expressed quantitatively or semi-quantitatively.

Risk management strategies

The risk management strategies aim to reduce the identified risks to a level of no concern, and considers defined areas of uncertainty. The risk management is described in terms of hazard and/or exposure reduction, and the consequent reduction in risk quantified when possible. Additionally, the reliability and efficacy of the measures used to mitigate the risks are assessed at this stage.

Overall risk evaluation

This is the overall risk evaluation of the GM plant taking into consideration the estimated risk, levels of uncertainty, knowledge gaps, assumptions made in arriving at the risk level, and the proposed risk management strategies. The overall risk evaluation results in informed (in qualitative or quantitative terms) guidance to risk managers. Justifications for why certain risks are acceptable are also provided at this stage, and may give rise to certain specific activities such as post market environmental monitoring.

In addition to the above six steps, the EFSA identified seven cross-cutting consideration and specific areas of risks to be addressed during ERA of GM plants (EFSA Panel on Genetically modified organisms (GMO), 2010).

Note: Steps in RA of food and feed from GM plants are described in EFSA Scientific Committee (2011).

Comparative safety assessment, as a general principle of risk assessment, is applied in both guidance documents. In risk assessment, hazards are defined as characteristics of the GM plants (or food and feed) which may cause adverse effects. Comparison is made to understand potentially harmful differences between a genetically modified plant (or food and feed) and the unmodified parent (or appropriate comparator).

In ERA and RAFF, risk assessment seeks to identify and characterize intended and unintended effects of genetic modification with respect to potential impact on environmental, human, and animal health. Data that can reveal these effects are derived from molecular characterization; compositional analysis; studies of interactions between genetically modified-plant and the environment as well as agronomic and phenotypic characterization.

For ERA, the process of correctly identifying potential hazards begins with systematic description of the case under assessment. Three components are considered, namely (i) the plant; (ii) the new trait and its intended effects as well as the phenotypic characteristics of the GM plant; and (iii) the receiving environment (Box 2, Step 1), which is when the scope of an ERA is defined. Scientific data to identify potential hazards, which are generated by practical testing of the GM plant, as well as the extent to which the receiving environment could be exposed to any identified hazard is estimated (Box 2, Steps 2 and 3) within the scope defined in Step 1. Resulting data are fed into subsequent steps to inform the overall outcome of ERA.

As stated in Box 2, EFSA has identified specific risk areas for which hazard characterization of a GM plant must be conducted, guided by specific protection goals (e.g., biodiversity conservation and ecological functions) formulated in Step 1, Box 2. Specific risk areas include persistence and invasiveness of the GMO, plant-to-plant gene flow, plant to microorganism gene transfer, interaction of the GMO with target organisms, interaction of the GMO with non-target organisms, impact of the specific cultivation, management and harvesting techniques, and effects on human and animal health (EFSA Panel on Genetically modified organisms (GMO), 2010). For example, hazard characterization in the risk area of “persistence and invasiveness” would require species-specific background knowledge of reproductive biology, weediness, invasive and persistence characteristics, hybridization and introgression potential with any compatible relatives. For viable propagating GM plants, i.e., GM plants that can germinate and thrive in the receiving environment, additional information according to a tiered 3-stage approach is required under current EU regulations.

The purpose of the information in stage 1 is to deduce whether the GM plant and its progeny can grow, reproduce and hybridize under the climatic and growth conditions of the specific receiving environment in the EU, and how the phenotypic characteristics (in particular growth and reproduction) compare to conventional counterparts. Answers to these questions are provided by collating information on seed germination characteristics, phenotype under agronomic conditions, reproductive biology and seed persistence (EFSA Panel on Genetically modified organisms (GMO), 2010). Information is further required in stage 2 for plants that can grow overwinter and/or can transmit genes to compatible relatives. The most direct way to answer this question is to conduct experiments in representative sites over a 2-year minimum period in the proposed receiving environment, as relative fitness is a function of environmental context (Birch et al., 2007). For GM plants with existing relatives or able to form feral population in the receiving environment, additional information is required in stage 3 to determine whether the GM trait confers fitness advantages to the GM plants, and whether the GM traits is capable of altering the fitness of compatible relatives or feral population in the new environment (EFSA Panel on Genetically modified organisms (GMO), 2010).

For RAFF from GM plants, hazard identification and characterization begin with molecular characterization of the GM plant. Molecular characterization is followed by comparative analysis of relevant characteristics of the GM plant and its comparator(s). The aim of these activities is to identify and characterize both intended and unintended effects on human and animal health (excluding other components of the ecosystem). The unintended effects may be due to genetic rearrangement or metabolite changes due to genetic modifications and can be detected by analysing the flanking regions of the inserts and by proteomic and/or metabolomic analyses of the end-product. Inserts are likely to affect known or predicted functions of endogenous genes. The EFSA guidance document requires in-depth information describing the identity of the nucleic acid intended for transformation, vector sequences potentially delivered to the GM plant, and characteristic of the DNA insert (EFSA Panel on Genetically modified organisms (GMO), 2011). In general, molecular characterization seeks to provide information on whether genetic modifications raise health concerns with regard to the interruption of endogenous genes, leading for example, to production of toxins, allergens, and/or anti-nutrients.

### Challenges for Risk Assessment of New and Emerging Gene-Editing Techniques

The present debate on how new and emerging gene-editing techniques will be regulated lacks a fundamental discussion on whether current risk assessment methodologies are adequate to analyze organisms arising from these techniques. A consequence of the recent ECJ ruling, which considers products of new and emerging gene-editing techniques as GMOs, is the question of adoption or adaptation of the current GMO risk assessment and risk management procedures for products arising from the new techniques. In this section, we look first at the potential challenges of adopting the current EU GMO framework for ERA of plants arising from the new directed mutagenesis techniques, and subsequently highlight the challenges of using the current guidelines for food and feed products from new techniques. We close with challenges to risk assessment in general, in particular with traceability and detection.

#### Environmental Risk Assessment

The current EU ERA framework was designed for GMOs produced via classical techniques of genetic modification (e.g., biolistic particle delivery or agro-bacterium mediated methods). Products of new and emerging techniques, according to the ECJ ruling, are all classified as GMOs, thus, raising a question of how the framework will be implemented. In particularly, an open question remains how to adapt guidance to support assessment of products arising from new and emerging gene-editing techniques (Lusser et al., 2011; EFSA Panel on Genetically modified organisms (GMO), 2012; Jones, 2015a; The Norwegian Biotechnology Advisory Board, 2018).

Given that a framework is only as good as its weakest elements, one strategy to determine the suitability of the current EU ERA framework for new directed mutagenesis techniques is to focus in particular on elements persistently critiqued by the scientific community (EuroActive, 2008; Hilbeck et al., 2011). Based on contemporary scientific critiques, the following elements of ERA of new directed mutagenesis techniques might be adopted or adapted: the focus of risk assessment; test-organisms; effect testing; post-release monitoring; and risk management.

##### The focus

Environmental Risk Assessment of new directed mutagenesis techniques may necessitate change in focus to include the entire crop plant, given that products of new and emerging gene-editing techniques may differ in complexity from conventional GM plants. This difference will depend on the extent of alterations engineered into a product using new techniques. At present and based on the concept of substantial equivalence, only change in trait or the newly expressed protein is emphasized in the implementation of the framework (Eu-Directive, 2001; European Commission, 2002). In addition, expansion of the scope of test compounds to include toxins and antitoxins may be necessary. Related, a lack of clear guidelines on cut-off, i.e., limit of concern, for substantial equivalence between GM- and non-GM plants is another element of test focus receiving critique (Millstone et al., 1999).

##### Test organisms

Choice of test organisms for evaluating target and non-target effects of products of new and emerging gene-editing techniques may necessitate a case-by-case selection of suitable testing species. Suitable testing species need to be representative of relevant ecological functions of the receiving environment, different from the current standard set of universal testing species that are representative of trophic levels of a generic ecosystem (OECD, 1981). This position has also been proposed as a remedy to the deficit inherent in the use of the current framework for ERA of GM plants (Hilbeck et al., 2011).

##### Effect-testing

In the current framework, where substantial equivalence is established, the stressor for which chronic effect, indirect effect and interaction effect testing is conducted is the new trait (e.g., an expressed protein or toxin, and not the whole plant) (Romeis et al., 2006, 2007). For products of new and emerging gene-editing techniques with a targeted knockout mutation, with no *a priori* known altered primary compound, no stressor may be identified, therefore no effect test can be deemed relevant. In this specific type of example, a focus on the entire GM plant also becomes necessary for robust effect-testing in ERA (Romeis et al., 2006, 2007).

##### Post-release monitoring

With new and emerging gene-editing techniques, it may be difficult to carry out post-release monitoring if similar mutations can also be found in conventional, not genetically modified varieties. This is a challenge unique to new and emerging gene-editing techniques, for example in the case of CRISPR/Cas9 where mutations involving a few nucleotide base-pairs which can also be achieved by conventional breeding techniques, or can occur naturally, is engineered into the target. For such products (especially SDN-1 category), it will be impossible to identify and associate the engineered modifications with a specific technique without prior knowledge of the type of modifications (or the techniques used to achieve the modifications). Thus, certain products of the new techniques of site directed mutagenesis cannot be detected, traced or monitored based on the requirements of the current framework, which needs the presence of marker sequences to identify a modified organism.

Many crops are changed using gene-editing techniques to delete various parts of target genes for either knocking out or change the gene functions. These crops are sometimes referred to as transgene-free crops, because even though the genetic composition is changed, no transgene DNA is integrated in the genome of these plants (Ricroch et al., 2017). The aim of deletion is most often elimination or changing the gene expression implicated with virus infections or other plant pests, rendering the crop more resistant to the particular infectious agent (Ricroch et al., 2017).

While advantageous for cultivated crops, such genetic changes may infer a huge selective advantage and thus create a high positive selection if pollen from cultivated fields are spread to wild relatives. Such gene flow is a major concern for GM crops and may be a realistic outcome of cultivation of disease resistant gene edited crops, unless co-existence measures are enforced (growing distance to wild population etc.). Since many genes have several functions, it is possible that knocking out or changing a specific gene function, may in addition to the intended effect, also alter unintended pathways. Assessing unexpected, unintended changes requires untargeted whole-genome profiling, post-release monitoring and general surveillance.

##### Risk management

In ERA, decisions of the risk managers are guided by the outcome of the scientific risk assessment (Box 2), which has risk management strategies as a part of the framework (Step 5, Box 2), where the Applicant outlines measures (including the reliability of the proposed measures) to reduce any identified risks. Therefore, if risk assessors lack experience evaluating the potential risks of new and emerging gene-editing techniques, this will reasonably impact the information provided to risk managers.

#### Risk Assessment of Food and Feed

Beyond the challenges with ERA of new and emerging gene-editing techniques listed above, current regulatory requirements are based on risk assessment developed and available when regulatory discussions on GMOs were just starting in the 1990s. It is therefore also necessary to discuss how to revise and adapt existing methods to better cover such challenges at the frontiers of biotechnology. Investigating the suitability of new methods implies assessing whether new tools, such as bioinformatics, and next generation sequencing or other -omics techniques, can complement or replace and thus contribute constructively to comparative assessment—or even to the assessment of whole gene-edited organisms when appropriate comparators are unavailable.

**FIGURE 3:**
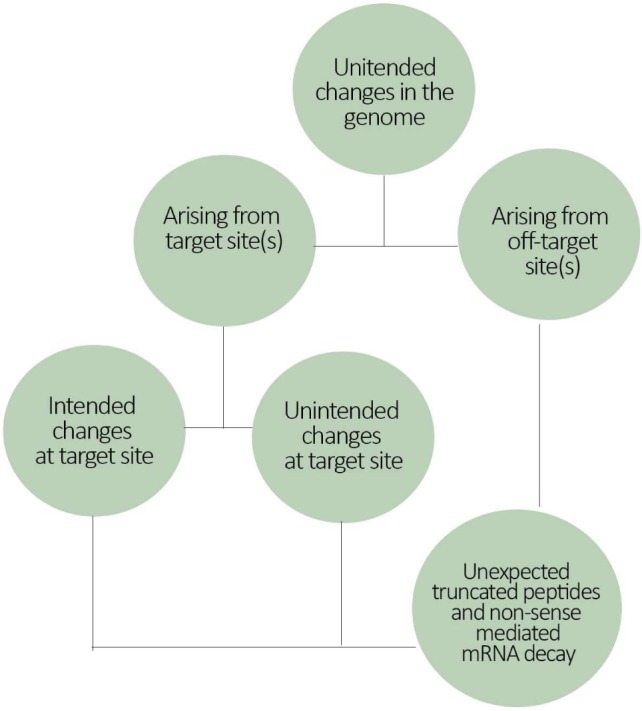
Flowchart illustrating the potential source of gene-editing mediated genome changes. Genome changes can vary in location (target and/or off-target sites), in quantity (how many sites were changed) and also in quality (deletion, insertion, substitution of nucleotides in a sequence). Therefore, genome changes at target site can have both intended and unintended effects depending on the quality/type of the change.

When it comes to new methods for RAFF, molecular characterization of a gene-edited organism may therefore need to take into consideration two main aspects of the genetic modification. One aspect is related to the spectrum of changes at the intended site (i.e., the nucleotide changes at target sequence). The second aspect refers to the spectrum of sites that have been changed. Both considerations are necessary because confining the change to the intended template only is not yet possible. Unintended effects might arise from both target site and off-target sites. Thus, after the procedure, intended products must be separated from unintended products (Figure 3).

#### Risk Assessment: Detecting Unintended Changes From New and Emerging Gene-Editing Techniques

Current EFSA guidelines for environmental risk assessment and risk assessment for GM foods and feeds start from identifying potential hazards associated with intended and unintended molecular changes. Potential hazards are assessed based on molecular description, comparative data with a non-GM counterpart followed by toxicological, allergenicity and nutritional assessments (EFSA Panel on Genetically modified organisms (GMO), 2010, 2011), as well as routine PCR and sequencing protocols and standard protein quantification protocols such as Western blots, ELISA testing or other spectrophotometry methods for assessing expression of newly introduced proteins (e.g., EFSA Panel on Genetically modified organisms (GMO), 2011; AHTEG, 2016). The idea behind hazard characterization and identification is to provide sufficient information on the description of the techniques used for the genetic modification, the source and characterization of nucleic acids used for transformation, nature and source of vector(s) used including nucleotide sequences intended for insertion, information on the sequences actually inserted/deleted or altered and the expression of the sequences as well as genetic stability of the inserted/modified sequence and phenotypic stability of the GM plant.

New and emerging gene-editing techniques might generate truncated polypeptides and/or non-sense-mediated mRNA decay either intentionally or unintentionally as part of a knockout process. Whereas such products are considered an unintended effect in transgene-based GMOs (Rosati et al., 2008), the desired phenotype in this case (i.e., resistance to a pest or an herbicide) is obtained by the nucleotide change in the gene of interest that generates the production of the non-functional gene products (Hussain et al., 2018).

*In silico* analysis can help identify mRNA variants and putative peptides derived from truncated DNA sequences or from potential read-through events, which should be then followed by *in vivo* RNA sequencing analysis. Characterizing peptide or protein variants is technically challenging because it relies on prior knowledge about binding sites to isolate the protein from an extract. If the binding site is lost or altered due to the genetic transformation, it means that this peptide variant will not be picked up for further analysis. If detected, it may not be fully distinguished from wild-type peptide variants that are also present in the sample. A recent review of detection methods for on-target changes generated by CRISPR and other sequence-specific nucleases is provided in Zischewski et al. (2017). As a specific example, MON810 and RR Soybean transgene cassettes have been found to produce read-through products which were further processed, resulting in four different RNA variants from which the transcribed region of the nopaline synthase terminator (tNOS) was completely deleted in soybean (Windels et al., 2001). In the case of MON810, RT-PCR performed in the 3′ end region of the transgene cassette produced cDNA variants of different length. An *in silico* translation of these transcripts identified 2 and 18 putative additional amino acids in different variants, all derived from the adjacent host genomic sequences, added to the truncated CRY1A protein with no homology with any known protein (Rosati et al., 2008).

Detecting unintended off-target changes can be more challenging than detecting changes at target sites because the number and position of nucleotide changes are unknown. There are also no data or guidance documents on test-methodologies to addresses unintended effects occurring due to off-target activity. If off-target effects occur within a gene, loss of gene function (truncation or gene deletion) or alteration of protein affinity or function (amino acid substitution) could be a possible outcome. Outside of protein coding genes, unintended alterations in promoters, introns or terminators could significantly alter gene expression. Plant allergens are also a major concern (Hoffmann-Sommergruber, 2000) and alterations of such allergens may constitute a health risk for human or animal consumption of plant foods. Screening for off-target sites at a genome-wide scale may be daunting, but in light of new directed mutagenesis techniques may be a necessary task for assessing the safety of commercialized products. A few approaches have been developed to investigate off-target activity of CRISPR modifications. These have been categorized into four major approaches: (i) *in silico* prediction, (ii) *in vitro* genome-wide assays, (iii) cell-based assays and (iv) *in vivo* screening.

*In silico* tools basically include all available software which have their own computational algorithms that identify likely off-target sites based on the sequence of the guide RNA. Pre-selected sites can be checked using the same methods described for target site detection and identification. Addgene’s team has created an online spread-sheet-based tool that compares these softwares and provides scores to each of their features so that a user can choose according to her or his needs. As a result, the tool generates a ranking of most suitable software (Addgene, 2017).

Many of the CRISPR/Cas9 design tools include information about potential off-target sites in the genome of interest, but it is important to keep in mind that not every algorithm searches for every kind of off-target effect (e.g., DNA or RNA bulges). It has also been observed that analyses from *in silico* predictions are not always correct and their results don’t always align because the CRISPR/Cas9 system is not completely understood (Zischewski et al., 2017).

*In vitro* and cell-based assays are mainly developed to search for CRISPR/Cas9 DSBs fingerprints. Digested genome sequencing, or Digenome-seq, is an *in vitro* assay that has become increasingly popular since its introduction in 2015 (Kim et al., 2015). Two newer methods are now also available, CIRCLE-Seq and SITE-Seq (Cameron et al., 2017; Tsai et al., 2017). Yet, these methods collectively remove genomic structural context. On the other hand, cell-based assays use different techniques to identify double-stranded breaks in genomic DNA within the cell environment. There are currently three approaches: BLESS (breaks labeling *in situ* and sequencing), GUIDE-Seq (genome-wide unbiased identification of DSBs enabled by sequencing), LAM-HTGTS (linear amplification-mediated high-throughput genome-wide translocation sequencing) (Crosetto et al., 2013; Tsai et al., 2015; Hu et al., 2016). However, Cas9 pharmacokinetic profile of the delivered components across cell and tissue types, especially the form factor of the gene editing components (DNA, RNA, or protein) and the delivery vehicle (viral or non-viral) is still a critical and underexplored determinant of Cas9 specificity (Tycko et al., 2016). Every time a different *in vitro* or cell-based assay is performed, a different off-target outcome might thus be expected. This potential variability makes it difficult to integrate across observations in a systematic, data-driven way. Consequently, these parameters are not taken into account by the majority of available off-target prediction tools.

Recently, the successful use of CRISPR in human cells has been connected to a selection process in CRISPR treated cells and shows that there may be other unique risk related factors to gene-editing, which are not discovered by searching for off-target DNA changes. Two papers showing that human polypotent stem cells that are treated with CRISPR may acquire mutations in P53 (Ihry et al., 2018) and immortalized human retinal pigment epithelial cells successfully treated with CRISPR may be exposed to a selection process against functional p53 (Haapaniemi et al., 2018). Even though these are experiments in human cells, the potential relevance for other species, including crops, should not be overlooked. The results may indicate that the successful integration of CRISPR edits could be impacted by genes connected to cell cycle arrest and DNA repair. If that is the case, the CRISPR induced selection of mutant cells may also occur in other species. A number of studies claim high precision and low to no off-target activity of CRISPR/Cas9 (e.g., Feng et al., 2018; Wei et al., 2018); however, whole genome sequencing (WGS) has recently documented off-target activity does in fact occurs in animals (Anderson et al., 2018) and plants (Braatz et al., 2017). When it comes to reducing off-target activity, gRNA design including RNA to DNA nucleotide replacements (Yin et al., 2018), length and composition of gRNA binding domain (Cho et al., 2014) as well as mismatched between gRNA and target DNA (Fu et al., 2014) seem to play a role. However, off-target activity has not been investigated to the extent of understanding thoroughly what governs changes outside the intended loci in the genome.

According to current understanding, the PAM (protospacer adjacent motif) sequence and its immediate upstream and downstream nucleotides, GC content of the gRNA, and epigenetics and chromatin structure of the target, each also have potential roles in off-target activity (reviewed in Jamal et al., 2016). Recently it has become evident that not only at which nucleotide CAS9 cuts, but also the sequence composition at the target, determines the outcome of the plant repair process (Vu et al., 2017). This indicates that not only gRNA binding but also the targeted sequence composition, will dictate factors such as the size of deletions and incorporation of mosaicism at the cut site.

## Discussion: the Need for New Molecular Characterization and Traceability Methods and a Responsible Research and Innovation Approach to Risk Governance of New and Emerging Gene-Editing Techniques

### Molecular Characterization and Traceability of New and Emerging Gene-Edited Plants

**FIGURE 4:**
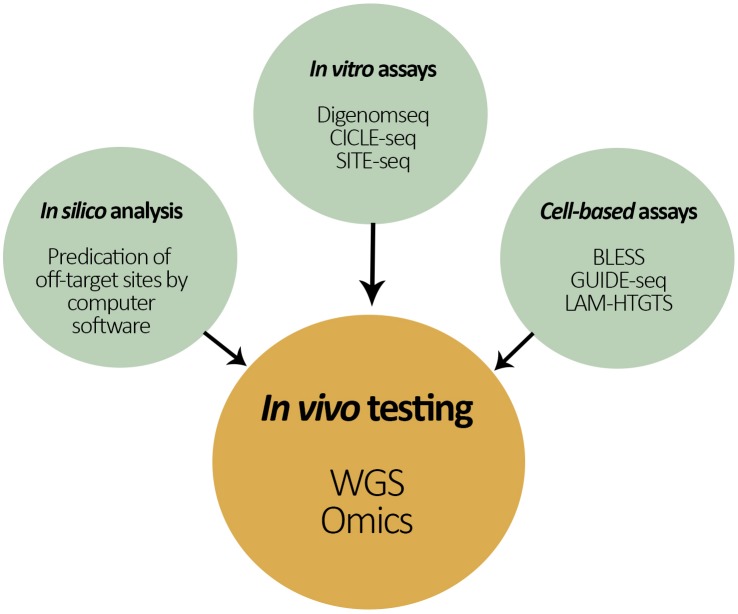
Schematic representation of the different approaches to test for unintended changes in the genome of a gene-editing organism. In the center is the proposed ‘*in vivo* testing approach’ by both whole genome sequencing (WGS) and ‘omics’ techniques for the assessment of off-target effects in the recipient’s genome.

Despite rapid progress of Cas9 specificity with marked improvements in guide RNA selection, protein and guide engineering, novel enzymes, and off-target detection methods; Cas9 protein still has been shown to bind and cleave DNA at off-target sites. To address the present limitations associated with *in silico* predictions as well as *in vitro* and cell-based testing of potential off-target sites, the ultimate unbiased method for measuring Cas9 off-target activity across the genome is WGS on the actual gene-editing organism (Figure 4). WGS provides a high-resolution, base-by-base view of the entire genome and is able to capture large and small variants that might otherwise be missed (e.g., if other targeted approaches were used). Consequently, WGS helps to identify potential unintended variants for examination in follow-on studies of gene expression and phenotypic analysis (Wang et al., 2018).

Whole genome sequencing strategies are based on high-throughput sequencing technologies such as Illumina dye sequencing, pyrosequencing, and SMRT sequencing. All of these technologies employ a basic shotgun strategy, namely, parallelization and template generation via genome fragmentation. More recently, nanopore sequencing has emerged as a new technique that performs “strand sequencing” in which intact DNA polymers through a protein nanopore, sequencing in real time as the DNA translocates the pore (Ambardar et al., 2016).

There are only a few studies that have applied WGS to investigate off-target activity of CRISPR *in vivo* systems. WGS has been applied for detecting off-target mutations by Cas9 in Arabidopsis (Feng et al., 2014), rice (Zhang et al., 2014), and tomato (Nekrasov et al., 2017). Unfortunately, these studies either looked only at potential off-target sites predicted by computer programs (bias analysis) or fell short of full analysis of all the mutations identified by WGS in edited plants (Tang et al., 2018). A recent paper investigated the degree to which GUIDE-Seq analysis predicted off-target changes by sequencing the whole genome of gene-editing mice embryos (Anderson et al., 2018). The results showed that 30 out of 43 off-target sites were predicted using a somewhat adapted version of GUIDE-Seq, meaning that remaining 13 off-target changes were not predicted and thus only detected due to the unbiased WGS approach performed.

Proper consideration of Cas9 genomic specificity for risk assessment should include not only the aggregate number of potential off-target sites for a given guide RNA, but also the physiological impact of individual off-target events (both detected or not) (Tycko et al., 2016). In particular, when it comes to hazard identification, characterizing the scope of off-target changes might not be enough to assess the potential adverse effects of gene-edited organisms. An evolving view of the use of omics techniques in addressing the biological relevance of molecular data is growing among risk assessors and regulators (Heinemann et al., 2011). ‘Omics’ techniques—for example proteomics, transcriptomics, metabolomics, etc.—are molecular profiling techniques used to screen for a certain type of molecule in a given sample and, thus, allow the simultaneous measurement and comparison of thousands of plant components without prior knowledge of their identity. There are different types of approaches to omics techniques, ranging from untargeted approaches (e.g., profiling all proteins present in a protein extract) to targeted approaches in which a specific feature in a class of molecules is targeted (e.g., screening and quantification of already known proteins) (Heinemann et al., 2011).

A combination of targeted and untargeted methods could allow a more comprehensive approach, and thus provide additional opportunities to identify unintended effects of new and emerging gene-editing techniques applied to plants. Different kinds of questions can be answered using profiling, as it can be used to identify and then characterize new molecules in a GMO (e.g., RNA, protein, metabolite) or molecules at very different concentrations (e.g., anti-nutrients). Profiling can also be used to detect pathological or other responses in a test organism that indicate an unintended change in the GMO and may also be useful for forming hypotheses to determine if the unintended changes were the cause of the adverse effects (Mesnage et al., 2016).

A recent initiative organized by EFSA in April was particularly interested in advancing ways of implementing omics techniques to current EFSA risk assessment guidelines.^4^ The outcome of this event, which drew on some 150 experts in the field, was supportive of the idea of adopting omics approaches toward risk assessment guidelines. In fact, EFSA started mapping the use of omics tools in the risk assessment related to food and feed safety back in 2014 (European Food Safety Authority [EFSA], 2014; EFSA Panel on Plant Protection Products and their Residues, 2017) but only recently started to build further toward a concrete path of implementation through guidance.

The regulation (EC) No 1830/2003 provides a framework for the traceability of GMOs and its products with the objectives of facilitating accurate labeling, monitoring the effects on the environment and the implementation of the appropriate risk management measures. This and the aforementioned regulations (Figure 1) established that GMO detection and identification methods have to be in place to allow GMOs to be traced and labeled. The method, which must be validated and published by the European Community reference laboratory established under Regulation (EC) No 1829/2003, is based on the detection of unique DNA sequences in the GMO. In other words, it must ensure the identification of the GM event and its reliable quantification. The framework and guidelines have been adequate to the GMOs being approved this far because they all contain the insertion of a foreign DNA sequence in a random genomic region. The variety of endogenous neighboring genomic sequences and the new transgenic DNA provided unique sequences that could differentiate each of the GMOs in the market to date.

However, as gene technology are developed it can be expected that a gene-edited organism containing one or a few nucleotide deletions or insertions at a specific genomic region might not be distinguished, at least using current methods, from a related variety or wild relative. This is because current GMO detection methods focus on a single DNA amplification sequence for its identification, i.e., the inserted and surrounding sequences. While specific methodologies to overcome this challenge will no doubt evolve since the decision of the ECJ, there are already developed plant variety and cultivar identification systems that can be adapted to gene-editing detection. The International Union for the Protection of New Varieties of Plants (UPOV) have refined biochemical and molecular techniques, as well as statistical tests and software for DNA-profiling which could serve as a basis for the gene-editing identification for both GMO traceability and labeling as well as for GMO patent rights (Korir et al., 2011). The main adaptation to this strategy from the previous GMOs methods is the need to perform more than a single DNA amplification test and the probability test to be conducted regarding the level of certainty of identification of a particular product. The presence of off-target DNA changes can also serve as a basis for the development of DNA amplification tests. In addition, plant variety and cultivar identification methods target the recognition of a single plant variety/cultivar while a gene-editing organism might be crossed to an infinitum of commercial crop varieties worldwide, which might then compromise referable results for gene-editing labeling.

Different strategies for DNA identification analysis, identity testing, profiling, and fingerprinting might have to be developed depending on the discrimination power that will be required by each gene-edited organism. Organisms with nucleotide insertions might have new and unique sequences that can be differentiated from any other species genotype using one or a few DNA amplification tests. While others, might require further sequencing tests of several DNA fragments.

### A Role for Responsible Research and Innovation Approaches to Formal Risk Governance Mechanisms

We have presented and reviewed a number of challenges related to risk governance of new and emerging gene-editing techniques. In addition to arguments for and against different types of regulatory frameworks we have, in light of the recent ECJ ruling, focused on limitations within current risk assessment approaches (ERA and RAFF alike). Beyond technological advances related to WGS and omics approaches for hazard and effect detection and monitoring, we have identified serval gaps in the knowledge base with regards to application of new and emerging gene-editing techniques to plants. In particular, we discussed knowledge gaps on the appropriate focus, selection of test organisms, and use of comparators when it comes to risk assessments of GMOs.

There are a diverse set of opinions on how knowledge gaps should be resolved in the application of new and emerging gene-editing techniques to plants in society. Risk analysis is value-based and “subjective,” meaning there is no absolute way for the process to move from scientific information to decision, despite more robust technical inputs such as from WGS or omics approaches. This issue relates to a classic example of an ill-structured “messy” challenge in science and technology policy (Metlay and Sarewitz, 2012), or a “post-normal science” issue (Funtowicz and Ravetz, 1993). The ways in which governments, industries, research institutes, and others decide to address thorny issues and knowledge gaps going forward is vital: what is chosen for knowing means also choosing what may remain unknown, and such intentional or accidental social production of ignorance will affect societal ability to assess, manage, and respond to social and environmental hazards (see for example Kleinman and Suryanarayanan, 2013).

Stepping back and presenting risk governance challenges in this way opens a larger conversation on what it means to responsibly research and innovate around agricultural biotechnologies (c.f., Hartley et al., 2016). Scholars and philosophers of scientific knowledge production have for decades been investigating such questions (c.f., Sismondo, 2008 for a review, and Kuhn, 1962; Winner, 1986; Laird, 1993 as specific examples). Broadly speaking, these communities recognize three overarching challenges related science and technology governance that make resolving the issues above a challenge (see von Schomberg, 2013; and Keeler et al., 2018): (i) why pursue research and innovation (orientation); (ii) who should be involved in research and innovation processes and why (legitimacy); and (iii) how to manage research innovation to achieve a desired outcome (control).

Recently, especially in Europe, the term “responsible research and innovation” (RRI) has come to describe a set of processes and outcomes intended to help resolve these general issues of science and technology governance in, with, and for society (see Stilgoe et al., 2013; von Schomberg, 2013; Foley et al., 2016 for a more detailed discussion on alignment of processes and outcomes for responsibility). Although it can first sound as if talking about responsibility means conversations about blame and accusation for ‘irresponsibility,’ the core of RRI conversations involve a set of questions directly related to the challenges like those we have presented with regulation and guidance of new and emerging gene-editing techniques: how to govern activities implicating existing, emerging, and new biotechnologies. In this context, a widely respected and accepted definition states that RRI is, “*A transparent, interactive process by which societal actors and innovators become mutually responsive to each other with a view to the (ethical) acceptability, sustainability and societal desirability of the innovation process and its marketable products (in order to allow a proper embedding of scientific and technological advances in our society)”* (von Schomberg, 2013, p. 64).

Responsible research and innovation approaches don’t presume to offer singular answers to scientific and societal questions. Instead, RRI encourages new ways of asking questions, exploring potential consequences of choices, and seeking answers when governing research and innovation activities. What is expected of benefits associated with application of new gene-editing techniques? How are intended and unintended effects to be assessed? When do assessed risks and promised benefits mean that a further research and innovation are justified? When not? What specific protection goals must be managed to avoid or mitigate unintended effects? As the Research Council of Norway, which strongly encourages adoption of RRI in its biotechnology funding, suggests: “Looking forward, thinking through, inviting along, and working together” (The Research Council of Norway [RCN], n.d.) can help address questions like the above associated with agricultural biotechnology risk governance.

National and international life sciences communities recognize the need for broader conversations about responsibility as well (c.f., The National Academies of Sciences, Engineering and Medicine [NASEM], 2016; Chneiweiss et al., 2017). Importantly, and as Hartley et al. (2016) have noted, a much broader community of people, organizations, experts, and interest groups will need to be involved in resolving questions like the above when approaching new and emerging gene-editing techniques through RRI. Evaluation on the state of RRI implementation in the Excellent Science Pillar of the EUR 77 billion eighth research and innovation (R&I) program highlights limited progress in adoption of inclusion of varied expertise in research and innovation activities (Bernstein et al., 2018). Indeed, beyond traditional industry and minimal civil society organization stakeholder engagement, engagement with non-traditional expertise in R&I is most commonly referenced as a unidirectional activity—for example, public outreach. In such one-way forms of “engaging” the public there is rarely opportunity for systematic reflection on or learning from diverse groups of people and expertise related, for example, to values associated with risks (Sturgis and Allum, 2004).

As a case in point specifically related to risk governance, we can return here to the challenge of communication between risk assessors and risk managers [a challenge recognized by EFSA in its 2016 guidance on specific protection goals for environmental risk assessment (EFSA Scientific Committee, 2016)]. EFSA guidance holds only that assessors and managers of risk are appropriate authorities to set specific protection goals (SPGs), identify stressors and hazards, and determine appropriate exposure pathways and adverse effects for risk assessment. On one hand, SPGs are defined as, “Explicit and unambiguous targets for protection extracted from legislation and public policy goals” (EFSA Scientific Committee, 2016, p. 9). On the other hand, the very approach that EFSA guidance states should be used to set these “explicit and unambiguous targets”—ecosystem services valuation—depends on complex, ambiguous, uncertain, and contested methodology (c.f., Millennium Ecosystem Assessment [MEA], 2005; Department for Environment, Food and Rural Affairs [DEFRA], 2007). This is not to say that attempting valuation beyond standard economic analysis is futile. Quite the opposite; but our point is to say that it is the very ambiguity and subjectivity of these environmental risk assessment processes that make an RRI approach so potentially useful (c.f., Funtowicz and Ravetz, 1993). From an RRI perspective, the scientific input into such processes is necessary but not sufficient: more diverse expertise and value-sets are needed to help respond to the ambiguity and contested-ness of risk governance challenges (Wickson and Wynne, 2012).

As Preston and Wickson (2016) have argued, new opportunities associated with contemporary approaches to genetic modification offer a chance to “improve governance through informing, shaping and guiding the actual development of emerging technologies (rather than just their regulation)” (p. 55). This chance is especially relevant with new and emerging gene-editing techniques because they are easy to apply, cheaper to use, and much faster than previous GM plant techniques, in addition to raising issues with potential detection, traceability, and labeling.

As we have noted above, efforts could be directed to improvements in regulatory guidance on ways that biosafety is studied and assessed. Wickson and Wynne (2012) identified needs for greater opportunities to enhance the robustness of independent scientific peer review of risk assessment dossiers; the transparency and openness with associated data; and the “time, resources, materials, and terms of reference” for independent biosafety researchers and advisors, “to perform the type of meaningful ‘independent assessments’ that such bodies are supposed to perform” (p. 335). These needs are greater today than ever, and directly related to responsible societal responses to addressing knowledge gaps arising from application of new and emerging gene-editing techniques in plants.

Beyond these scientific questions, the regulatory science and broader life science, biotechnology, and other communities associated with GM plant risk governance can look to other societal actors for help. Policy communities have long experience and expertise with developing processes to combine scientific, political, and public inputs into decision-making about science and technologies (c.f., Metlay and Sarewitz, 2012). Industries have vested interests in supporting responsible and inclusive approaches (to demonstrate concern for safety, beyond profit, and retain their permissions from society to operate) and are very adept at adapting their research, production, and wider value-chains to societally determined guidelines [c.f., research on the idea of companies seeking and working to keep a “social license to operate” (Moffat et al., 2016)]. Social and political scientists have a breadth of expertise, theories, and tested methods for engaging heterogenous and diverse groups of people on controversial and important topics, and the role of scientific experts in this process (c.f., Pielke, 2007). Humanists, artists and philosophers have perhaps the deepest traditions of grappling with and constructively raising the types of questions humans unearth at the expanding frontiers of engineering life. We agree with the conclusions of Hartley et al. (2016) that when it comes to enhancing guidance in current GMO regulations, the way forward will require, “commitment to candor, recognition of underlying values and assumptions, involvement of a broad range of knowledge and actors, consideration of a range of alternatives, and preparedness to respond” (p. 2 of 7) to support responsible use of gene-editing in plants with and for society.

## Conclusion

The current risk assessment framework was developed for products of classical GM techniques. As the 25 July 2018 ECJ decision points out, new and emerging gene-editing techniques lack a long-history of safe-use in any organism. Indeed, the scientific literature reveals the biotechnology community is still focused, at a fundamental level, on improving the efficiency and the applied uses of such techniques. Given this reality, a key question for the field going forward is not whether to regulate for safe use of biotechnologies, but how.

Several aspects of the current framework and its implementation stand to benefit from reconsideration in light of progress in the broader field. Examples of these aspects include: choice of test organisms for identification of target and non-target effects; use of the whole edited plant/derived-product as stressor in effect-testing; and expansion of the repertoire of molecular techniques to include omics in molecular characterization of hazards (Ramon et al., 2014; Casacuberta et al., 2015). In particular, the risk assessment guidance may need to be revised to enhance suitability for evaluating impacts of products by new and emerging gene-editing techniques on environmental, human, and animal health.

The present moment offers an opportunity to advance GM plant risk governance anchored in biosafety research and RRI approaches. This is especially true as the ECJ reminds the field of biosafety approaches structured by the guiding European Union application of the precautionary principle. Considering such approaches points to the need for more, better resourced, transparent, and independent risk assessment of products of gene-editing techniques, intended and unintended effects, as well as target and off-target activity.

Responsible research and innovation, the Commission’s approach to applying the precautionary principle in research and innovation funding (Bourguignon, 2015), is well suited to supporting risk governance of the complex, value-laden issues associated with gene-edited plants. Through an RRI approach—supplemented with technological advances of WGS and omics approaches—could involve a broader community of people, organizations, and interest groups when reflecting on, anticipating, and responding to risk governance challenges (Baltimore et al., 2015). In pursuit of broader societal consensus, the scientific community came together to use its moral authority and remain in control of the pace of research on inheritable changes in human germ lines (c.f., Wade, 2015). The broad plant-biotechnology community could similarly explore more open and coordinated pursuit of societally desirable, ethically acceptable, and sustainable changes to plant life, grounded in principles of biosafety.

## Author Contributions

AM provided a first draft of this article and wrote the regulatory section. AO wrote the environmental section. SA-T wrote the food and feed and molecular characterization and traceability section. O-GW wrote the post release monitoring section. MB wrote the RRI section and led on English-language revision. All authors contributed to the final draft of this manuscript.

## Conflict of Interest Statement

The authors declare that the research was conducted in the absence of any commercial or financial relationships that could be construed as a potential conflict of interest.

## Footnotes

1 Norwegian Gene Technology Act is available in English at: https://www.regjeringen.no/en/dokumenter/gene-technology-act/id173031/

2 The Convention on Biological Diversity Clearing House Mechanism platform at https://www.cbd.int/chm/ and the Biosafety Clearing House platform set up by the Cartagena Protocol on Biosafety at https://bch.cbd.int/.

3 Provisional text of the ECJ ruling is available in English at: http://curia.europa.eu/juris/document/document.jsf?text=&docid=204387&pageIndex=0&doclang=EN&mode=req&dir=&occ=first&part=1&cid=709582#Footnote$^{*}$

4 EFSA Colloquium on omics techniques for GMO risk assessment. Available at: https://www.efsa.europa.eu/en/events/event/180424-0.
